# Nasal Polyposis and Serum Albumin: Systemic Effects of Local Inflammation

**DOI:** 10.7759/cureus.34859

**Published:** 2023-02-11

**Authors:** André De Sousa Machado, Francisco Rosa, Ana Silva, Luis Meireles

**Affiliations:** 1 Otolaryngology, Centro Hospitalar Universitário do Porto, Porto, PRT; 2 Otolaryngology, Hospital das Forças Armadas, Porto, PRT

**Keywords:** nasal polyps, inflammation, local inflammation, serum albumin, nasal polyposis, chronic, nasal, sinusitis, nose, albumin

## Abstract

Introduction

The genesis of chronic rhinosinusitis is always a topic of debate. A polyp is a pale, edematous tissue emerging mostly from the middle meatus. An epithelial injury caused by continuous inflammation of the nasal mucosa is considered to be a possible cause of the genesis of nasosinusal polyps.

Objective

To understand the link between serum albumin levels and nasal polyposis.

Methods

A retrospective study of 180 consecutive patients between January 2016 and January 2020 at our center. We then divided the patients into three age- and gender-matched groups: 60 patients with chronic rhinosinusitis with nasal polyposis, 60 patients with chronic rhinosinusitis without nasal polyposis, and a control group with 60 patients. No patient had a history of any pathology that could alter serum albumin. We then compared the level of serum albumin between the three groups.

Results

The group of patients with rhinosinusitis and nasal polyposis consisted of 60 patients with a serum albumin value of 4.49 ± 0.29 g/dL, whereas in the control group, the serum albumin value was 4.67 ± 0.2 g/dL. We found a significant difference between the group with nasal polyposis and the other two groups evaluated: chronic rhinosinusitis without nasal polyposis (p<0.001) and the control group (p<0.001).

Conclusions

Lower levels of serum albumin can be seen in patients with chronic rhinosinusitis with nasal polyposis. Further studies should aim to apply its value since it is a non-expensive marker, to the follow-up of those patients or even to stratify them according to their endotype.

## Introduction

The European Position Paper on Rhinosinusitis and Nasal Polyps (EPOS) considers chronic rhinosinusitis (CRS) as an inflammation of the nose and paranasal sinuses with the presence of two or more of the following symptoms for a duration of more than 12 weeks: nasal obstruction, nasal discharge, facial pressure or pain, and hyposmia [1]. For the diagnosis, it is also necessary to use a paranasal CT scan or nasal endoscopy, which will also determine the phenotype of chronic rhinosinusitis with or without nasal polyps (CRSwP or CRSsP) [1].

Nasal polyps are inflammatory lesions that project into the nasal airway and have their origin in the nasal mucosa [2-5]. Nasal polyps are typically bilateral [1]. The etiology of nasal polyposis is still under debate. The term "polyp" is used to refer to a pale and edematous mass, mostly with an origin in the middle meatus. According to Larssen et al., the epithelial damage caused by local inflammation plays a major role in the genesis of nasal polyps [5].

Albumin is the most abundant protein in plasma, corresponding to between 50 and 60% of the proteins present in the plasma compartment, with 30% to 40% found in the intravascular compartment [6]. One of the most important functions of albumin is the maintenance of the capillary pressure balance with the maintenance of an osmotic and hydrostatic pressure gradient at the level of the capillary microcirculation, regulating the flow of interstitial liquid at this level. Albumin is responsible for 70%-80% of capillary osmotic pressure. The decrease in albumin concentration leads to a decrease in the osmotic pressure, with consequent leakage of fluid from the blood capillaries and consequent tissue edema [6].

Albumin also acts as a negative inflammatory marker; inflammatory diseases cause a decrease in plasmatic concentration. The factors that contribute to this are related to an increase in vascular permeability and an increased rate of local cell renewal with the consumption of albumin. Also, due to inhibition via cytokines (interleukin 4 (IL-4), IL-5, IL-6, and IL-13), less albumin is synthesized [1, 7, 8].

Since EPOS 2020, there has been increasing experimental data supporting the fact that the nasal epithelium is the primary portal of entry for respiratory viruses as well as an active component of initial host responses against viral infection. The cascade of inflammation initiated by nasal epithelial cells will lead to damage by the infiltrating cells, causing edema, engorgement, fluid extravasation, mucus production, and sinus obstruction in the process [1].

In this study, we aim to search for a link between the local and systemic inflammation in CRSwNP, i.e., between albumin levels and CRSwNP.

## Materials and methods

Study design

A retrospective study was carried out in our center, Centro Hospitalar Universitário do Porto, in Porto, Portugal, to review the clinical files of patients who met the criteria for inclusion and also those of patients in a control group.

Chronic rhinosinusitis was first medically treated according to the literature, and if the persistence of symptoms or a decrease in quality of life were detected, surgical treatment was proposed [1]. The diagnosis of nasal polyposis was made after the removal of masses of paranasal sinuses by functional endoscopic sinus surgery (FESS) and an anatomopathological study [1].

The study was conducted according to regulations established by the Helsinki Declaration.

Study population

The inclusion and exclusion criteria are stated below.

Inclusion criteria: age between 18 and 60 years; diagnosis of rhinosinusitis; who underwent FESS between January 2016 and January 2020; anatomopathological diagnosis.

Exclusion criteria: Previous nasosinusal surgery, incomplete files, and patients with pathologies or problems likely to alter levels of serum albumin, such as oncology therapy, systemic inflammatory diseases (Crohn's disease, celiac disease), hepatic or renal pathology, malnutrition, and dehydration.

A control group without nasosinusal pathology was created (consisting essentially of patients undergoing stapedotomy and type I tympanoplasty), without any relevant medical background, pathologies, or treatments that could alter serum albumin levels.

Variables evaluated

The following variables were evaluated: sex, age, nasal symptoms, degree of polyposis (when present), the extent of FESS, procedures associated with FESS, and serum levels of albumin. The control group underwent a complete ear, nose, and throat examination; individuals who had no pathology were included in the study.

All patients with nasal pathology underwent previous medical treatment. Patients were administered oral corticosteroids and antibiotics for seven days preoperatively.

The evaluation of polyposis degree was performed by anterior rhinoscopy with a rigid endoscope (4 mm, Storz-0 °, Germany) after decongestion and local anesthesia. The Johansen scale was used to stratify nasal polyps (degrees 1-3) (Table 1) [9].

**Table 1:** The Johansen's scale [9]

0	No polyps
1	Polyps are restricted to the middle meatus (mild polyposis).
2	Polyps expand beyond the middle meatus without going beyond the lower border of the inferior turbinates (moderate polyposis).
3	Polyps expand beyond the lower border of the inferior turbinates (severe polyposis).

A CT of the paranasal sinuses was mandatory for all the patients who presented with nasal pathology before surgery.

If the possibility of a septoplasty existed, the procedure started with decongestion of the nose and infiltration of lidocaine with epinephrine (1% lidocaine with 1:100,000 epinephrine). Through the extension of the inflammatory process, uncinectomy, maxillary antrostomy, anterior and posterior ethmoidectomy, and enlargement of the natural os of the sphenoid sinus and frontal sinus were performed. In patients with nasal polyposis, the surgery included its removal, which was confirmed after anatomopathological analysis [10].

Blood sample collection

Venous blood samples were taken from all patients and sent to the lab. Values of serum albumin between 3.2 and 5.6 mg/dL were considered normal values for the population [11].

Statistical analysis

All analyses were performed using IBM Statistical Package for Social Sciences (SPSS) version 28 (IBM Corp., Armonk, NY), and p-values below 0.05 were considered statistically significant. A descriptive analysis of patient characteristics was performed, considering absolute and relative frequencies (for categorical variables) and mean and standard deviation (for continuous variables). The normal distribution was checked using Kolmogorov-Smirnov, and Levene's test was used to check the homogeneity of variances in each group. The comparison between groups was performed using the analysis of variance (ANOVA) test after the normality of the data was verified. A p-value < 0.05 was considered statistically significant.

## Results

Three groups were created, to which we assigned the letters A, B, and C. Group A consisted of the group of patients with an anatomopathological and endoscopic diagnosis of CRSwNP: 60 patients (38 men and 22 women) with an average age of 51.6±12.25 years (Table 2).

**Table 2:** Characterization of groups A, B, and C and respective serum albumin values in the groups CRSwP: chronic rhinosinusitis with nasal polyposis; CRSsP: chronic rhinosinusitis without nasal polyposis

	Number	Age ± SD (years)	Gender		Mean value (g/dL)	Standard deviation
			Male	Female		
Group A (CRSwP)	60	51.6 ± 12.25	38	22	4.49	0.29
Group B (CRSsP)	60	49,03 ± 14.29	28	32	4.64	0.33
Group C (control group)	60	47,13 ± 9.75	28	32	4.67	0.2

At the endoscopic examination, the patients in group A had polyps obstructing the middle meatus bilaterally. The most common degree of nasal polyposis according to the Johansen scale was II in 45% of patients (Table 3).

**Table 3:** Distribution of patients according to the degree of nasal polyposis [9]

Degree of nasal polyposis (Johansen scale)	%
I	25
II	45
III	30

Those patients presented with recurrent nasal obstruction (N=52), rhinorrhoea and hyposmia (N=47), and facial pain or pressure (N=35). The main complaint of the patients with CRSwNP was nasal blockage (87%), followed by anterior rhinorrhea (47%) (Table 4).

**Table 4:** Symptoms in patients with chronic rhinosinusitis with nasal polyposis

Symptoms	Patients with CRSwNP (N/%)
Nasal blockage	52 / 86,6%
Rhinorrhoea	47 / 78,3%
Pain or facial pressure	35 / 58,3%
Anosmia or hyposmia	22 / 36,7%
Nasal itching or sneezing attacks	7 / 11,7%

Group B consisted of the group of patients with chronic CRSsNP: 60 patients (28 males and 32 females) with an average age of 49,03±14.29 years (Table 2).

All patients with nasal pathology (groups A and B) underwent FESS; medium meatotomies (100%) and anterior ethmoidectomy (80,8%) were the most frequent procedures performed in 81% of the patients (Table 5).

**Table 5:** FESS extension in patients with chronic rhinosinusitis FESS – functional endoscopic sinus surgery

Procedure	N/ %
Medium meatotomy	120 / 100%
Anterior ethmoidectomy	97 / 80.8%
Posterior ethmoidectomy	70 /58.3%
Frontal sinusotomy	25 / 20.8%
Sphenoidectomy	18 /15%

48.3% of patients underwent septoplasty, and 24.2% underwent a medium partial turbinectomy (Table 6).

**Table 6:** Surgical procedures associated with FESS in patients with chronic rhinosinusitis FESS: functional endoscopic sinus surgery

Procedure	N/ %
Septoplasty	58 / 48.3%
Medium partial coronectomy	29/ 24.2%
Turbinoplasty of concha bullosa	20/ 16.6%
Inferior partial coronectomy	16/ 13.3%

Group C (the control group) consisted of patients without nasal pathology, consisting essentially of patients who underwent stapedotomy and type I tympanoplasty; it was composed of 60 patients (28 men and 32 women) with an average age of 47.13 ± 9.75 years (Table 2).

It was found that the age value of the population can be assumed to be normally distributed in the three groups to take (Kolmogorov-Smirnov test, p = 0.983 for group A; p = 0.253 for group B; and p = 0.776 for group C); homogeneity of the age variances of the three groups can also be assumed (Levene's test, p = 0.421), which allowed us to compare the means of the three groups with an ANOVA test (F (2,177) = 3.2; p = 0.12) and we didn’t find a statistically significant difference between groups.

The serum albumin value was 4.49 ± 0.290 g/dL in patients in group A, 4.64 ± 0.33 g/dL in group B, and 4.67 ± 0.2 g/dL in group C. According to Kolmogorov-Smirnov and Levene's test, we found in three groups values of significance > 0.05, which allowed us to compare the means of three groups with an ANOVA test (F (2,177) = 47.376; p = 0.001), and we found a statistically significant difference in serum albumin value between groups. From the paired comparisons using the Tukey test, statistically significant differences were found between the means of serum albumin between groups A and B (p = 0.002) and between groups A and C (p = 0.001); no statistically significant differences were found between the means of serum albumin values between groups B and C (p = 0.984) (Table 2).

## Discussion

In our study, we found that the albumin value, in the groups assessed with age and gender without statistically significant differences between them, was higher in patients with CRSwP and CRSsP, as well as among patients with CRSwP and patients without nasal pathology.

Recent evidence shows CRSwP as a different endotype of rhinosinusitis [12]. Although, what we know is that it is mostly a T helper type 2 (Th2) inflammatory response (with amplification of cytokines like IL-4, IL-5, and IL-13). In contrast, CRSsNP has more diverse immunological endotypes that are not well defined but are characterized by a distinct inflammatory pattern with a type 1 inflammation endotype mediated by interferon-gamma (IFN - γ) and neutrophils [13, 14].

Edematous stromal tissue and the formation of pseudocysts are common features of nasal polyposis, and it has been suggested that the retention of plasma proteins, such as albumin, may contribute to its growth [14].

Recently, some insight has been given into the mechanisms that may drive the formation of nasal polyposis and implies some role for serum proteins, in which we include albumin [15,16].

Remodeling of sinonasal tissues in chronic rhinosinusitis (CRS) is the basis of polyp formation, goblet cell hyperplasia, and other anomalies that account for some of the disease's symptoms. When barrier remodeling is considered, the result is greater permeability of the nasal mucosa, making the persistence or recurrence of CRS easier. These phenomena previously described are most common in type 2 CRS, possibly contributing to greater symptomatology in these patients and a higher rate of relapse. The link between the endotype and the remodeling pattern is still under study [1].

In CRSwNP, we see a global disruption of the epithelium in nasal tissue samples with excess mucus being produced; this can be explained by goblet cell hyperplasia and mucin hypersecretion [14,17,18].

It was found that the value of albumin was lower in patients with CRSwNP and CRSsNP, as well as among patients with CRSwP and patients without nasal pathology. Age and gender didn’t act as confounders between groups.

Karatas et al. conducted a similar study to this one; however, we added another group of comparison (CRSsP) besides the control group with no nasal pathology. With this, we can, with all the cautions, take some notes about the role of serum albumin levels in the pathogenesis of nasal polyposis and not only in chronic rhinosinusitis per se [19].

As limitations of this study, we consider the limited number of elements in each group, the retrospective nature of the group, and the presence of possible factors that can alter serum albumin levels, namely nutritional status and type of diet. Local biopsies should also be performed in order to add more power to the findings.

Even though we consider our findings to be in consonance with the literature, a decrease in the serum albumin value is seen in patients with CRSwNP.

The difference in albumin values can help further characterize the patient's endotype. A cut-off that can reliably distinguish the different endotypes, allowing therapeutic optimization, can be seen as a possibility with the emergence of endotype-directed therapy in the treatment of CRSwNP. A differential diagnosis of sinonasal neoplasms should also be made [20].

A limitation of the study can be the fact that serum immunoglobulin E (IgE) and eosinophilic count weren't taken into account, and those variables can also play a role in nasal polyposis.

The authors also consider it relevant in the future to carry out studies that allow the association of serum albumin levels with the time of evolution of chronic rhinosinusitis, ideally, prospective cohort studies.

## Conclusions

In this study, we saw that lower levels of serum albumin can be seen in patients with CRSwP. Further studies should aim to maximize its value, since it is a non-expensive marker, on follow-up of those patients or to stratify them according to their endotype and aim for the best possible treatment of this chronic and prevalent pathology; also, these findings might contribute to a further understanding of the pathophysiology of chronic rhinosinusitis.
